# Investigation of *Morinda citrifolia* Activities through Pinoresinol and α-EG Related Gene Expression

**DOI:** 10.3390/plants11151985

**Published:** 2022-07-30

**Authors:** Runglawan Sudmoon, Sanit Kaewdaungdee, Unchaleeporn Ameamsri, Tawatchai Tanee, Pornnarong Siripiyasing, Warin Wonok, Arunrat Chaveerach

**Affiliations:** 1 Faculty of Law, Khon Kaen University, Khon Kaen 40002, Thailand; rsudmoon@yahoo.com; 2 Department of Biology, Faculty of Science, Khon Kaen University, Khon Kaen 40002, Thailand; sanit_k@kkumail.com (S.K.); unchaleeporn.a@kkumail.com (U.A.); warin1104@hotmail.com (W.W.); 3 Faculty of Environment and Resource Studies, Mahasarakham University, Maha Sarakham 44150, Thailand; tawatchai5@hotmail.com; 4 Faculty of Science and Technology, Rajabhat Mahasarakham University, Maha Sarakham 44150, Thailand; psiripiyasing@gmail.com

**Keywords:** α-EG, pinoresinol, *Morinda citrifolia*, *Morus alba*, qRT-PCR, *COL1A1*, *COL1A2*, *COL3A1*, *FGF1*, *FGF7*

## Abstract

α-EG is a unique substance that was first found in the leaves and fruits of *Morinda citrifolia* (Mc) growing in Thailand using GC-MS at 52.33% and 54.12%. It was then concentrated and its abundance quantified, along with that of pinoresinol, via GC, compared to the standards in leaves, ufp, rfp, rawfs, and seeds. α-EG and pinoresinol, which have collagen stimulating, skin whitening, and an inhibitory effect on wrinkle formation, were found in different concentrations and amounts. Three different concentrations of the five Mc part extracts were tested on *NHDF* for gene expression related to the aforementioned activities, *COL1A1*, *COL1A2*, and *COL3A1*, *FGF1* and *FGF7* by qRT-PCR. The results showed various expression levels, both stimulatory and inhibitory, with different concentrations of plant parts and genes. Similar results were revealed when the experiments were performed with *Morus alba* (Ma), which was found to contain 20.48 g protein p/100 g leaves at concentrations of 3.11 mg/mL. The studied Mc parts seem to have advantages based on the stated objectives, gene type and level of activity of each plant part. Rawfs and leaves supplemented with Ma samples were selected for toxicity tests with PBMCs. The lack of both cell and DNA toxicity from the rawfs indicated that they can be used safely.

## 1. Introduction

Plants benefit humanity in many ways. For example, *Morinda citrifolia* (Mc) is a useful pantropical species supporting human life as an edible fruit; it is also used to create drinks, supplements, etc., and has medicinal value. There are many publications on it in the literature. Various aspects of the species have been studied, including its chemical and medicinal properties, and new reports and reviews are being published all the time. Some important Mc studies have indicated the the plant’s fruit has anti-inflammatory, anti-cancer, and antioxidant properties. Additionally, a 50% ethanolic noni seed extract showed more potent in vitro inhibition of elastase and tyrosinase, 1,1-diphenyl-2-picrylhydrazyl (DPPH) radical scavenging activity than leaf extract [1]. Xeronine, a small alkaloid which is one of the bioactive compounds of noni fruit, is believed to be capable of modifying the molecular structure of specific inactive proteins, thereby regulating the folding of active enzymes [2]. It has also been reported that the plant has anti-inflammatory effects which may be useful in the treatment of inflammatory bowel disease [3]. Furthermore, the consumption of noni fruit juice does not cause any significant disturbances to the liver or kidney outside of immune modulation [4]. The main alkaloids present in noni are xeronine, scopolenin, dammacanthol; it also contains vitamins A and C, Mg, K, Fe, alizarin, and ursolic acids, and therefore, may be able to slow the growth of cancer. Additionally, it may be a viable therapeutic resource for the treatment of obesity and obesity-related metabolic problems or indigestion [5]. Drinking 40 mL per day of noni wine was shown to effectively prevent high-fat diet-induced oxidative stress and obesity in mice [6]. Noni fruit water extract and noni fruit polysaccharide were shown to alleviate oxidative stress and inflammation in mice on a high-fat diet, and the noni fruit polysaccharide at 100 mg per kg bodyweight had a better effect than noni fruit water extract with a similar polysaccharide dosage, illustrating that noni fruit polysaccharide may be an important component in the water extract [7]. *M. citrifolia* fruit juice contains large groups of secondary metabolites including polyphenols, reducing compounds, mucilage and terpenoids. The antimicrobial activity of these compounds was found to inhibit the growth of *Staphylococcus aureus*, *Pseudomonas aeruginosa*, *Proteus mirabilis*, *S. epidermidis*, *Proteus vulgaris*, *Streptococcus oralis*, *Enterococcus faecalis* and *Escherichia coli* [8]. Additionally, four publications reported important data about the species. Noni seeds contain 3,3’-bisdemethylpinoresinol and americanin A, which have been found to inhibit tyrosinase activity. Inhibited α-melanocyte activity stimulates melanogenesis, resulting in a skin whitening effect, wrinkle inhibition and enhanced blood fluidity, platelet aggregation and fibrinolytic activity [9]. Several phytochemical constituents which contain the most valuable compounds for industrial and pharmacological activities have been described [10]. Additionally, the phenolic acids, lignans, flavonoids, flavones, flavans-3-ol, anthocyanins, phytosterols, alkaloids, vitamins and minerals present in the noni plant have been shown to affect obesity and obesity-associated metabolic dysfunction by various mechanisms [11]. In [12], the traditional uses, phytochemicals, phytotherapic properties, toxicological properties and recent advances in the processing and standardization of products derived from noni fruit were reported. The advantages of this species seem to be attributable to the abundance of chemicals that give rise to its therapeutic properties. One other plant species with high nutritional value, i.e., mulberry, *Morus alba* (Ma), is a well-known plant food for animals such as silkworm. Its leaves and fruits are also widely consumed by humans and have reported medicinal properties. The authors of [13] studied the safety of consuming the leaves and proposed a pharmacological dosage of a leaf extract, which might be a valuable drug [13]. Currently, mulberry leaves are authorized as a food resource with high protein, carbohydrate, vitamin, microelements and dietary fiber contents. They are also rich in phenolic acids, flavonoids, alkaloids, and γ-aminobutyric acid [14]. As such, their use has been proposed as a food supplement.

Another area of ongoing research is how different environments may produce different substances and properties in *M. citrifolia*, since the plant grows all over the tropics and has a myriad of uses. As such, researchers need to undertake further study on the substances, activities and genes that determine these characteristics in the leaves, raw and ripe fruit, and seeds to confirm and augment the data that have already been published. In this research, the protein matter in Ma will be studied to clarify whether it supports gene expression.

## 2. Results

### 2.1. Macronutritional Components

According to a proximate analysis, 100 g plant leaf powder of Ma leaves comprised 16.91 g ash, 316.46 Kcal energy, 11.10 g moisture, 20.48 g protein, 45.81 g total carbohydrate and 5.70 g total fat.

### 2.2. Phytochemical Constituent Evaluation by Gas Chromatography-Mass Spectrometry

**Figure 1:**
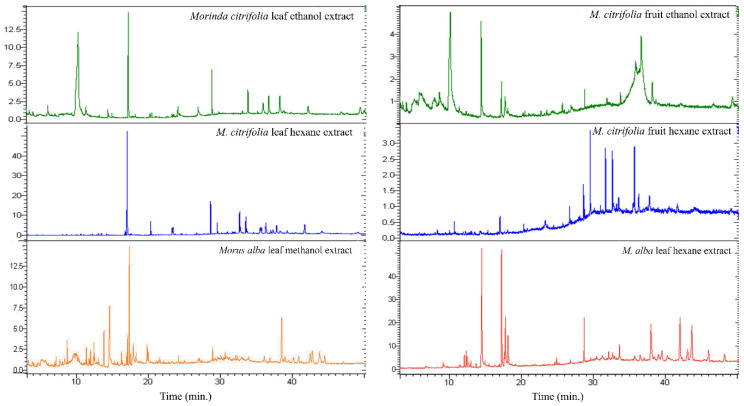
Gas chromatography-mass spectrometry chromatograms of the hexane and ethanol leaf extract of *Morus alba* (Ma) and leaf and fruit extracts of *Morinda citrifolia* (Mc), showing retention time and peak areas.

Mature Ma leaves and the leaves and fruits of Mc were analyzed. The various types and quantities of detected phytochemicals are listed in Table 1, and chromatograms showing retention time and peak areas are shown in Figure 1. The major chemical in Mc parts is ethyl-α-d-glucopyranoside or α-EG, which made up 54.12% and 52.33% of the ethanol extracts of leaves and fruits, respectively. This compound was not found in the Ma leaf extract.

### 2.3. Gas Chromatography (GC) Compared to the Pinoresinol and α-EG Standards

**Figure 2:**
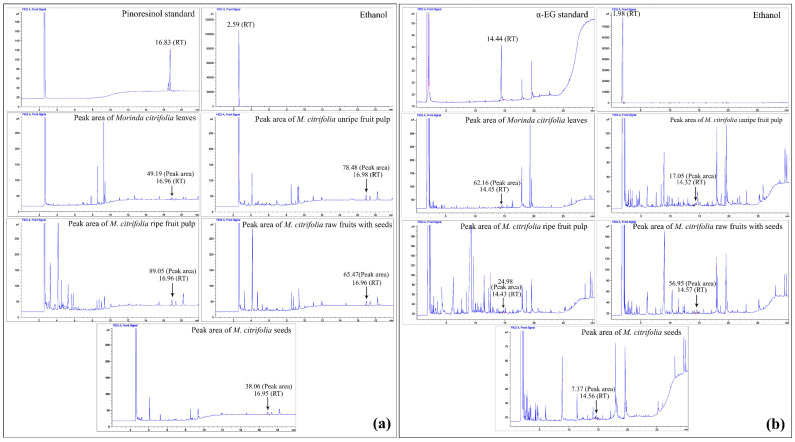
GC chromatograms of the pinoresinol (**a**) and α-EG standards (**b**), ethanol solvent and pinoresinol and α-EG peak areas from *Morida citrifolia* (Mc) plant parts.

The abundances of 3,3′-bisdemethylpinoresinol (pinoresinol) and ethyl-α-d-glucoside (α-EG) were measured in five Mc parts, i.e., the leaves, ufp, rfp, rawfs, and seeds. Pinoresinol was found in all studied plant parts, with the highest abundances in both concentrations of rfp being 0.14 mg/mL and 0.38 mg/g (Table 2), based on a peak area in the extract chromatograms (Figure 2). The plotted graph of the peak areas and the pinoresinol standard concentration created a linear equation, i.e., y = 749.07x − 18.13, and a correlation coefficient (R^2^) of 0.9991. α-EG was also found in all studied plant parts, with the highest concentration in leaves, i.e., 0.30 mg/mL, and in rawfs, i.e., 3.77 mg/g plant sample (Table 2, Figure 2). The plotted graph of the peak areas and the α-EG standard concentration created a linear equation, i.e., y = 239.75x − 10.004, and the correlation coefficient (R^2^) was 0.9965.

### 2.4. Gene Expression Quantification

**Figure 3:**
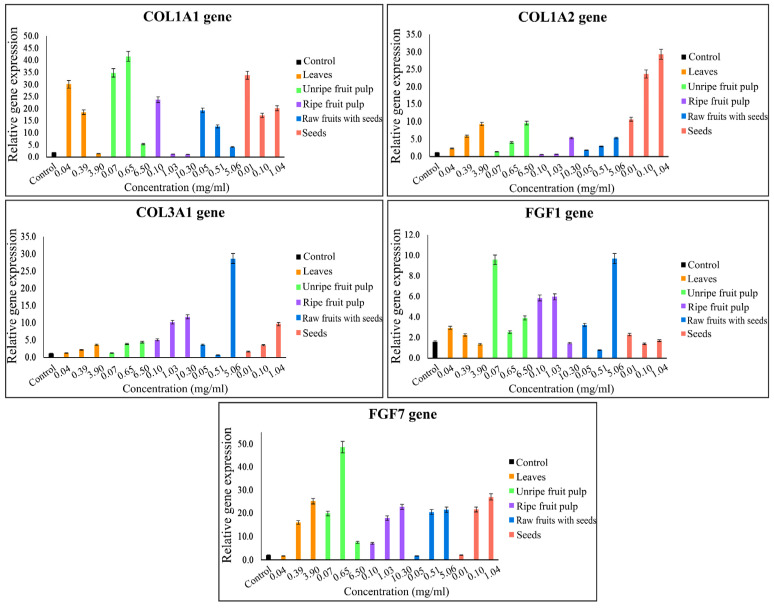
Graphs plotting the stimulatory gene expression value and the studied plant parts with various concentrations, showing the stimulatory effect of *Morinda citrifolia* (Mc) parts/extracts containing α-EG and pinoresinol.

The results of the gene expression research are divided into two parts. The first part presents the results regarding genes which should be expressed in high percentages in plants and which are effective at stimulating type I collagen, *COL1A1* and *COL1A2*, type III collagen and *COL3A1*, as well as angiogenesis-stimulating and fibroblast proliferation genes *FGF1* and *FGF7*, which were quantified by quantitative reverse transcription-polymerase chain reaction (qRT-PCR). Three concentration levels of the samples were tested. The results indicated that all studied plant parts and their extract concentrations affected all studied genes expressed in various high percentages compared to a control (without the plant extracts). Graphs of various relative gene expression and the studied plant parts with various concentrations are shown in Figure 3. The observed increases in expression percentages were as follows: *FGF1* and *FGF7* genes increased by 970% from 5.06 mg/mL rawfs, followed by 960% from 0.07 mg/mL ufp, 4870% from 0.65 mg/mL ufp and 2700% from 1.04 mg/mL seeds. The expression percentages of collagen stimulation genes were 4160% and 3480% from 0.65 mg/mL and 0.07 mg/mL ufp, respectively, and 3380% from 0.01 mg/mL seeds for *COL1A1* collagen type I; 2930%, 2370%, and 1070% from 1.04, 0.10, and 0.01 mg/mL seeds, respectively, and 960% from 6.50 mg/mL ufp for *COL1A2* collagen type I; finally, 2870% from 5.06 mg/mL rawfs and 1180% from 10.30 mg/mL rfp for *COL3A1* collagen type III.

**Figure 4:**
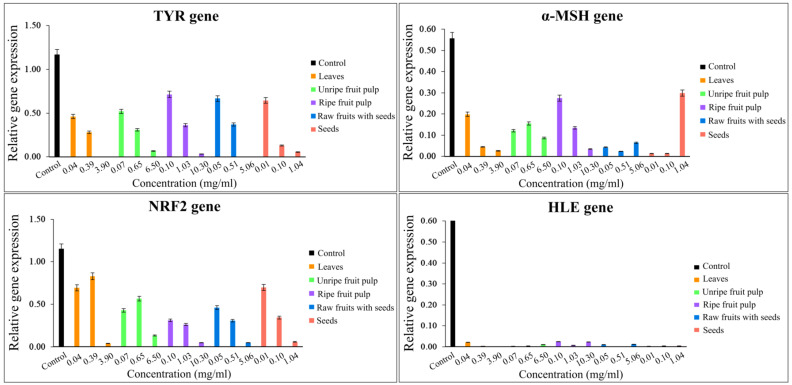
Graphs plotting the inhibitory gene expression value and the studied plant parts with various concentrations, showing the inhibitory effect of *Morinda citrifolia* (Mc) parts/extracts containing α-EG and pinoresinol.

The second part contains the results regarding genes which should be expressed in low percentages but with high inhibitory efficacies, as follows: *TYR*, an essential enzyme in melanin synthesis; *α-MSH*, an α-melanocyte-stimulating hormone gene which stimulates melanogenesis; and *NRF2*, a transcription factor for stress response. These factors cause dull skin and aging, and therefore, should not be highly expressed. The results indicated that all studied plant parts and concentrations affected all of these genes compared to a control (without the plant extracts). Graphs of various relative gene expression levels and the studied plant parts with various concentrations are shown in Figure 4. The lowest and second lowest percentage expression which showed high efficiency were 0.10% from 5.06 mg/mL rawfs, followed by 0.20% from 3.90 mg/mL leaves for the *TYR* gene; 1.29% from 0.01 mg/mL and 1.33% from 0.10 mg/mL seeds, followed by 2.33% from 0.51 mg/mL rawfs for the *α-MSH* gene; 3.84% from 3.90 mg/mL leaves, followed by 4.70% from 10.30 mg/mL rfp for the *NRF2* gene; 0.20% from 0.51 mg/mL rawfs, and 3.90 mg/mL leaves, followed by 0.30% from 0.01 mg/mL seeds, 0.065 mg/mL ufp and 0.39 mg/mL leaves for the *HLE* gene.

**Figure 5:**
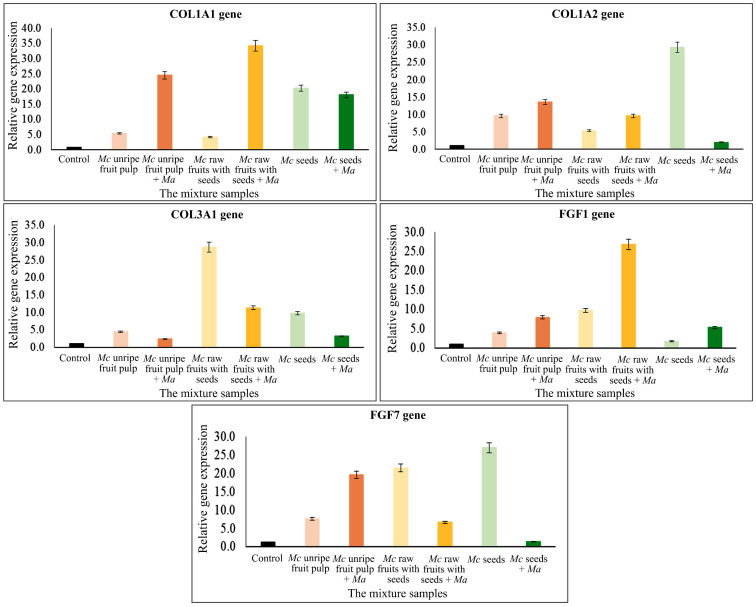
Stimulation gene expression values and the mixture of plant parts with various concentrations of *Morinda citrifolia* (Mc) containing α-EG and pinoresinol, and *Morus alba* (Ma) leaves containing 20.48 g total protein per 100 g at a concentration of 3.11 mg/mL.

**Figure 6:**
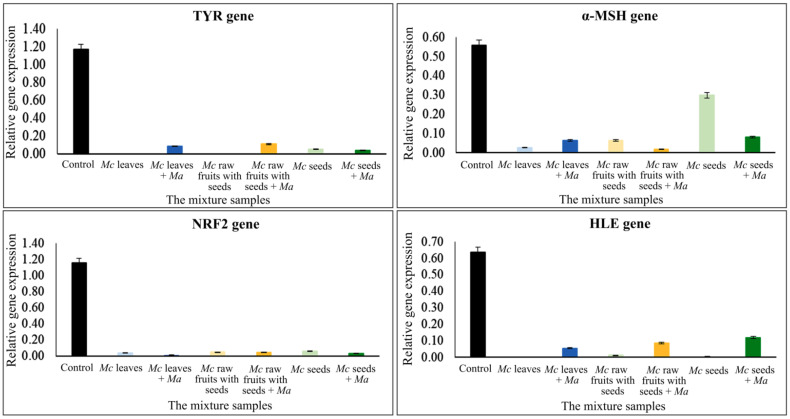
Inhibition gene expression values and the mixture of plant parts with various concentrations of *Morinda citrifolia* (Mc) containing α-EG and pinoresinol, and *Morus alba* (Ma) leaves containing 20.48 g total protein per 100 g at a concentration of 3.11 mg/mL.

**Figure 7:**
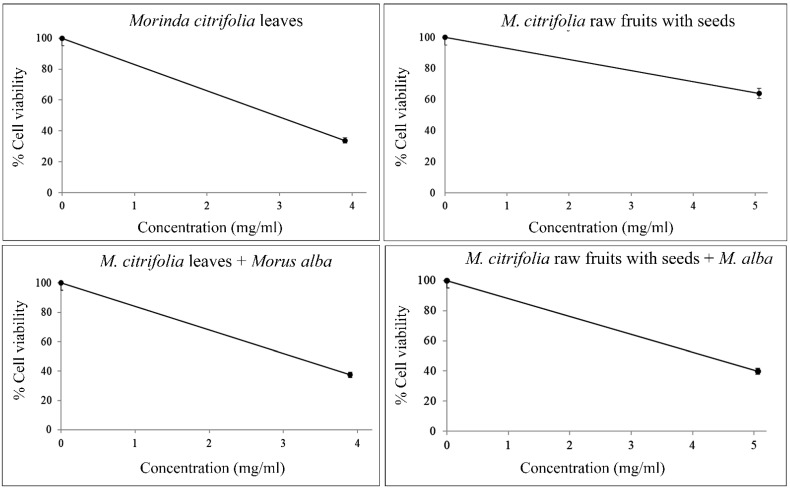
Cytotoxicity showing viability percentages of PBMCs treated with ethanol extracts of *Morinda citrifolia* leaves, *M. citrifolia* rawfs, *M. citrifolia* leaves plus *Morus alba* and *M. citrifolia* rawfs plus *M. alba* extracts.

The gene expression levels of the mixed extracts, comparing the three Mc parts, i.e., ufp, rawfs and seeds (containing α-EG and pinoresinol), and *Morus alba* (Ma) leaves containing protein were tested. The result showed that there were various levels of expression depending on the kind and concentration of Mc parts and genes. Additionally, both higher and lower expression were observed when Ma was added; for example, percentage expressions were 3200% and 2450% from 5.06 mg/mL rawfs and 6.50 mg/mL ufp for the *COL1A1* gene, i.e., higher than without Ma. However, with1.04 mg/mL seeds, higher expression, i.e., 2930%, was observed without Ma for the *COL1A2* gene. All results are shown in Table 3 and Figure 5. The results for the inhibitory genes showed that all studied mixtures, i.e., Mc leaves, rawfs and seeds with and without Ma, affected expression to a lower extent than with and without Ma, as shown in Table 4 and Figure 6. Rawfs at a concentration of 5.06 mg/mL and leaves at a concentration of 3.90 mg/mL without Ma showed lower activity, i.e., 0.10% and 0.20% for *TYR* and *HLE*, respectively, but showed higher activity, i.e., 0.19% and 1.40%, at a concentration of 5.06 mg/mL rawfs and 3.90 mg/mL leaves with added Ma for *α-MSH*, *NRF2*. With various activity found from the Mc parts, leaves and rawfs, the mixtures of Mc leaves and Ma leaves and rawfs were further tested for toxicity at a working concentration, as described in Table 5, Figure 7.

### 2.5. Cytotoxicity and Genotoxicity Testing via MTT and Comet Assays

**Figure 8:**
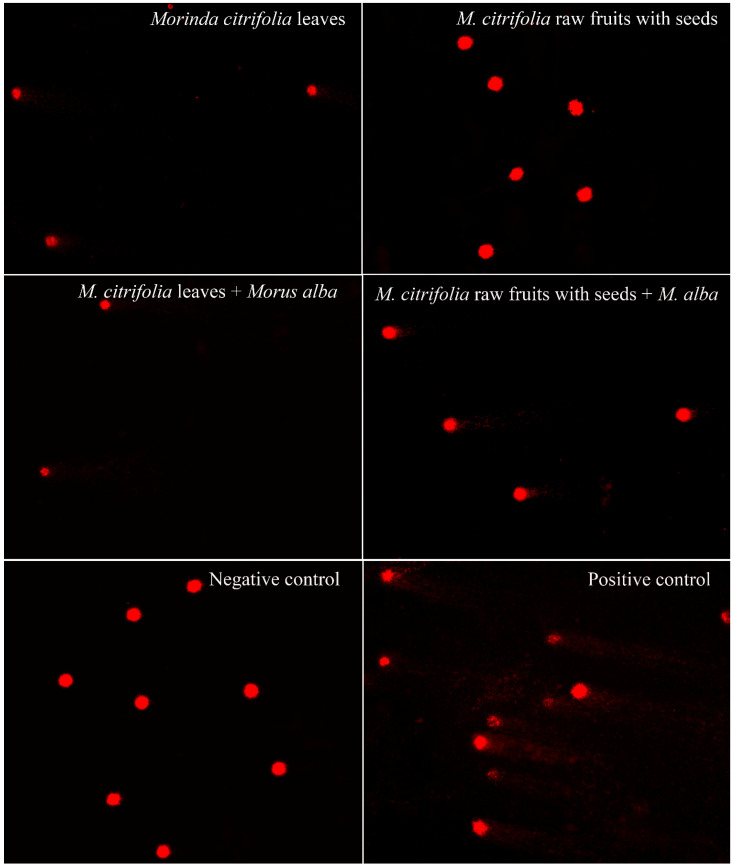
Comet images (200×) of PBMCs treated with ethanol extract of *Morinda citrifolia* leaves, *M. citrifolia* rawfs, *M. citrifolia* leaves plus *Morus alba*, *M. citrifolia* rawfs plus *M. alba* extracts; negative and positive controls.

The results of a MTT assay (3-(4,5-dimethylthiazol-2-yl)-2,5-diphenyltetrazolium bromide assay) showed IC_50_ values at 3.00 mg/mL, 2.90 mg/mL and 3.60 mg/mL in Mc parts and leaves, and in the mixture of Mc and Ma leaves. Following depth toxicity testing on the DNA level by comet assay, the samples showed significant DNA damage (Table 5, Figure 8). With the LD_50_ value calculated using IC_50_ values, the toxicity was revealed as Class II, i.e., slightly hazardous, corresponding to over 2000 mg/kg body weight for rats by oral or dermal application.

## 3. Discussion

When analyzing substances using the GC-MS method, α-EG was not found in Ma. Therefore, the quantity and concentration of this substance were not further studied.

The first finding regarding α-EG in Mc (grown in Thailand) leaves ufp, rfp, rawfs, and seeds provided very important information following the discovery of pinoresinol [9]. The research results can be applied for the creation of natural products including traditional forms, modified forms like supplements, nutraceuticals, functional foods, and cosmetics. α-EG acts to stimulate collagen production; collagen is an extracellular matrix protein, i.e., the major component of the extracellular matrix of the dermis. It accounts for 90% of the fiber component. Thus, change in dermal collagen content greatly affects dermal homeostasis [15]. Collagen produced by the body is synthesized mainly by fibroblasts, i.e., the main structural protein found in skin, tendon and bone. Additionally, collagen has been found to have many pharmaceutical, medicinal, nutritional and cosmetic applications [16,17]. Pinoresinol, which is present in Mc fruit, has several functions. It was tested and shown to have tyrosinase inhibitory activity in melanogenesis, an inhibitory effect on matrix metalloproteinase-1 (MMP-1) secretion (MMP-1 is a major collagenolytic enzyme which is responsible for collagen damage) and an inhibitory effect on wrinkle formation by *HLE* creation, which degrades collagen I and elastic fiber in human skin. Thus, *HLE* inhibition may be useful for the prevention of wrinkle formation [9].

On this basis, human normal fibroblast was selected in this study for experiments to show the expression of angiogenesis and fibroblast proliferation genes *FGF1* and *FGF7*, type I collagen genes *COL1A1* and *COL1A2*, and type III collagen gene *COL3A1*. Fibroblasts produce substances that constitute the basic structure of skin; a decrease in their proliferation and activity causes the deterioration of the skin structure [18]. The activities of inhibiting *α-MSH*-stimulated melanogenesis and an inhibitory effect on MMP-1 secretion (*HLE* activates MMP-1) mean that it is expected that these activities and the two substances, α-EG and pinoresinol, will be useful for the prevention of photoaging and wrinkle formation, as well as collagen creation and skin whitening. However, the amount and concentrations in each studied Mc part should be clarified. From the studied parts, leaves ufp, rfp, rawfs and seeds, and differences (concentrations in mg/mL and amounts in mg/g sample) contained 0.30/1.51, 0.11/1.75, 0.15/2.48, 0.28/3.77, and 0.07/0.94 α-EG and 0.09/0.20, 0.13/0.34, 0.14/0.38, 0.11/0.28 and 0.08/0.20 pinoresinol. These values do not reveal, however, whether the concentrations and quantities are sufficient to stimulate and inhibit the effects of the aforementioned genes. Therefore, mRNA gene expression was measured by qRT-PCR from the total RNA extracted from extracts treated with NHDF. The result showed strong effects compared to the control, with expression stimulation levels of up to 3480–4160%, 2930% and 2870% from ufp and seeds for type I and III collagen COL1A1, COL1A2, COL3A1, and 2700–4870% from seeds and ufp for angiogenesis and fibroblast proliferation genes FGF1 and FGF7 (Table 4). The inhibitory expression showed low levels but high efficacy in rawfs at 0.10% for the TYR gene, seeds at 1.29% α-MSH, and rawfs and seeds at 0.20–0.30% HLE. Additionally, leaves also showed low percentages but high efficacy for inhibition of TRY, i.e., 0.20%, and of NRF2, i.e., 3.80%. The NRF2 gene regulates oxidative stress and toxicity [19]. In line with expectations, if the protein substrate from Ma leaves was added, gene expression in the stimulatory (*COL1A1*, *COL1A2*, *COL3A1*, *FGF1* and *FGF7*) and inhibitory groups showed various higher or lower expressed values for different genes. Similarly, with and without Ma leaves, *TYR* and *HLE* showed higher or lower expression. This indicated that leaves and ufp, as well as rawfs, have the potential to stimulate and inhibit the expression of all of the studied genes. However, the other studied Mc parts seemed to have advantages, depending on the objective, gene type and plant part used. Given this, the applications of Mc parts including leaves, rawfs and these two parts combined with Ma leaves at a ratio 6:4 passed toxicity tests, both in cell and DNA by MTT and comet assays. The overall results showed no toxicity in rawfs.

As such, the raw fruit with seeds can be safely applied in natural products, i.e., both foods and cosmetics. However, IC_50_ values indicated that these three parts should be used within limits, because of the observed Class II toxicity [20].

## 4. Materials and Methods

### 4.1. Plant Materials and Extract Preparation

The plant species were collected from a field in Muangkhonkaen District, Khon Kaen Province, Thailand and were identified by a proficient botanist, prof. Arunrat Chaveerach, Ph.D. Permission was not required to collect the *Morus alba* and *M. citrifolia* materials used in this study, because they are common species which grow widely in gardens, fields and forests for domestic consumption, i.e., eating the leaves and fruits by drinking and cooking. Specimen numbers will be granted 2 years after the deposition. Mature leaves of *Morus alba* and mature leaves and fruits of *M. citrifolia* were collected, washed and air-dried, and then ground into a powder. The powder was combined with hexane or ethanol, separately at a rate of 1:5, and soaked for 72 h. Each solution was filtered through a Whatman no.1 filter paper. The filtrates were kept at −20 °C until used in experiments, including phytochemical component analysis by GC-MS and for GC comparisons.

### 4.2. Proximate Analysis of Nutritional Content

The air-dried *Morus alba* leaf samples were ground to a fine powder. The powder was employed for proximate analyses at the Central Laboratory (Thailand) Co., Ltd., Muangkhonkaen, Thailand following the standard protocols of the Association of Analytical Chemists (AOAC) [21].

### 4.3. Gas Chromatography-Mass Spectrometry (GC-MS)

Phytochemical analyses of *M. alba* and *M. citrifolia* were performed using an Agilent Technologies GC 6890 N/5973 (Santa Clara, CA, USA) inert mass spectrometer fused with a capillary column (30.0 m × 250 μm × 0.25 μm). Helium was used as the carrier at a constant flow rate of 1 mL/min. The injection and mass-transferred line temperature was set at 280 °C. The oven temperature was programmed to increase from 70 °C to 120 °C at 3 °C/min. It was then maintained isothermally for 2 min, and subsequently raised to 270 °C at 5 °C/min. A 1 μL aliquot of the extract was injected in split mode. The relative percentages of the extract constituents were expressed as percentages using peak area normalization. Components were identified by comparing the obtained mass spectra with reference compounds in the Wiley 7N.1 library.2.4.

### 4.4. Ethyl-α-d-Glucoside (α-EG) and 3,3′-Bisdemethylpinoresinol (Pinoresinol) Detection by GC Compared to the Standard

Ten-gram samples of *M. citrifolia* including leaves, ufp, rfp, rawfs and seeds were soaked in 50 mL ethanol (at a ratio of 1 g:5 mL), and the solution was kept in a dark room for 3 days. The mixture was filtered through Whatman no.1 filter paper (125 mm diameter) and fractioned by adding a mixture of ethyl acetate and dichloromethane (at a ratio of 1:2). The separated clear mixture was precipitated by adding a solution mixture of petroleum ether:diethyl ether (ratio 2:1) for 400 µL per 1 µL sample, until the solution precipitated. The clear solution was filtered by syringe filter, with pore sizes of 0.45 µm, and each final extract was used for α-EG and pinoresinol detection.

The chromatographic conditions for pinoresinol detection were as follows: the GC was an Agilent Technologies GC7890B (Santa Clara, CA, USA) equipped with a flame ionization detector (FID) and HP-5 capillary column (30.0 m × 320 μm i.d. × 0.25 μm film thickness) (Agilent, Santa Clara, CA, USA). Helium was used as the carrier gas at a flow rate of 1 mL/min. The injector and detector temperatures were 260 °C and 320 °C, respectively. The oven temperature was programed at an initial temperature of 120 °C and a ramp rate of 10 °C/min for a final temperature at 300 °C, which was maintained for 10 min. Then, 1 μL of each sample was injected into a column.

The chromatographic conditions for α-EG detection were almost identical, but with the following differences: the injector and detector temperatures were 250 °C and 300 °C, respectively; the oven temperature was programed at an initial temperature of 50 °C, a ramp rate of 10 °C /min and a final temperature at 250 °C, which was maintained for 10 min. Subsequently 1 μL of each sample was injected into a column.

Standard preparations of pinoresinol and α-EG. First, 1 mg of pinoresinol and α-EG each were diluted with 1 mL methanol. The working solution was prepared by serial two-fold dilutions yielding six concentrations. These various concentrations were applied to plot the calibration curve. The linear equation and correlation coefficient were calculated using Microsoft Excel.

### 4.5. Gene Expression Analysis by qRT-PCR

#### 4.5.1. Extract Preparation

The five plant parts were washed, air-dried for 2–3 days and then ground into powder. A 20-g sample powder each was extracted using 100 mL of 70% ethanol (at a rate of 1 g:5 mL). The mixture was filtered with Whatman no. 1 filter paper, and the filtrate was evaporated by rotary evaporator (Rotavapor R-210, Buchi, Flawi, Switzerland) at 800–1000 mbar, 15 °C, and 600 rpm for 2 h before being re-dissolved with 10% dimethyl sulfoxide (DMSO). The solution was 10-fold diluted with distilled water to obtain three working concentrations.

#### 4.5.2. Gene Expression Quantification

1. Cell activation

*NHDF* (ATCC, Manassas, VA, USA) was cultured in 24-well plates at a primary concentration of 1.7 × 10^5^ cells/well in modified low glucose Dulbecco’s Modified Eagle Medium (DMEM) medium supplemented with 10% FBS and 1% antibiotic (streptomycin and penicillin). It was then incubated at 37 °C and 5% CO_2_ for 24 h.

2. Gene stimulation and inhibition

After removing the medium, DMEM containing 1% FBS was added. The cultured cells were added to the sample extract at 200 µL/well and incubated at 37 °C and 5% CO_2_ for 48 h. The medium was removed, the cells were washed with PBS, and the cells were total RNA extracted using a GF-1 RNA extraction kit (Vivantis, Shah Alam, Malaysia). The RNA quality and quantity were checked using a nanodrop spectrophotometer (MaestroGen, Hsinchu, Taiwan) at 260/280 nm. The RNA quantity for all samples was normalized and 10 µg of the RNA samples was used. Reverse transcription for the first strand cDNA was performed using a Viva 2-steps RT-PCR kit with M-MuV RT (Vivantis, Shah Alam, Malaysia) by reverse transcriptase and oligo (dT)18 primers (Vivantis, Shah Alam, Malaysia). The reaction mixture was incubated at 65 °C for 5 min, and the reaction was terminated by heating 85 °C for 5 min. cDNA was kept at −20°C until further gene expression measurements were taken.

The cDNA was the template for the amplification reaction of expression detection by qRT-PCR (Roche, Mannheim, Germany) using gene-specific primers. The primer sequences were (5′→3′) *COL1A1* F-AGCCTCTCCATCTTTGCCAGCA and R-GATTCCCTGGACCTAAAGGTGC [22], *COL1A2* F-CCTGGTGCTAAAGGAGAAAGAGG and R-ATCACCACGACTTCCAGCAGGA [22], *COL3A1* F-TGGTCTGGAAGGAATGCCTGGA and R-TCTTCCCTGGGACACCATCAG [22], *FGF1* F-ATGGCACAGGTGGATGGGACAAG and R-TAAAAGCCCGTCGGTGTCCATG [22], *FGF7* F-CTGTCGAACACAGTGGTACCTG and R-CCAACTGCCACTGTCCTGATTTC [22], *GAPDH* F-GTCTCCTCTGACTTCAACAGCG and R-ACCACCCTGTTGTGTAGCCAA [22], *TYR* F-CCATGACAAATCCAGAACCC and R-GGACTAGCAAATCCTTCCAG [23], *α-MSH* F-GTCCTCAGAAAGCTTCCTTTCCGC and R-CGTTGCCTGGAAACCGCGGCGTCTC [24], *NRF2* F-TTCTGTTGCTCAGGTAGCCCC and R-TCAGTTTGGCTTCTGGACTTGG [25], *HLE* F-GCTGGAGCCCTCAGTTGTGGG and R-TGTTGGGTAGAATGGTGCCGG [26]. The 20 µL mixture reaction contained 5 µL 4× CAPITAL qPCR green mix LRox (Biotech rabbit, Berlin, Germany), 0.5 µL forward primer and 0.5 µL reverse primer (10 µM), 12 µL PCR grade water and 2 µL cDNA. The thermal cycles were programed following pre-denaturation at 95 °C for 3–5 min, followed by 45 cycles at 95 °C for 15 s at 60–62 °C for 30 s and 72 °C for 30 s, followed by cooling at 40 °C for 30 s. The melting temperature curve was analyzed for specific- and non-specific PCR product separation. When the reaction was complete, the crossing point for gene expression evaluation was found by the 2^−∆∆Ct^ method [27].

List of primers for gene stimulation and inhibition detection by qRT-PCR technique including stimulation genes: *COL1A1*, *COL1A2* are type I collagen genes; *COL3A1* is type III collagen gene; *FGF1* and *FGF7* stimulate angiogenesis and fibroblast proliferation; the inhibition gene Tyrosinase (*TYR*) is an essential enzyme in melanin synthesis; *α-MSH* is α-melanocyte-stimulating hormone gene, stimulating melanogenesis; finally, *NRF2* is a transcription factor for stress response. The genes were determined relative to *GAPDH*.

3. Protein plant extracts affect gene expression

*M. citrifolia* parts were selected for the experiment (including concentrations of the plant parts of 6.50 mg/mL ufp, 5.06 mg/mL rawfs, and 1.04 mg/mL seeds by gene expression for stimulation, and 3.90 mg/mL leaves, 5.06 mg/mL rawfs and 1.04 mg/mL seeds for inhibition) with a protein extract of *Morus alba* at a concentration of 3.11 mg/mL (derived from the first extract concentration in 100% DMSO diluted to the working concentration, 10% DMSO) with a substrate expected to promote gene functions, i.e., stimulating the mechanism of collagen creation by *COL1A1*, *COL1A2* and *COL3A1*; and angiogenesis and fibroblast proliferation by *FGF1*, *FGF7*. The *Morus alba* leaf protein extract contained 20.48 g total protein per 100 g, in accordance with the standard protocols of the Association of Analytical Chemists [28]. The three studied powders were mixed with *Morus alba* leaf powder at a ratio of 60:40. Then, the mixed powder was passed through the extract preparation and gene expression was quantified as described. Gene expression analyses were performed by qRT-PCR (Roche, Mannheim, Germany).

### 4.6. Cytotoxicity and Genotoxicity Testing via MTT and Comet Assays

The plant sample parts which had high expressions of collagen genes and inhibited tyrosinase gene expressions according to the aforementioned primers were selected for toxicity testing, i.e., both cytotoxicity (3-(4,5-dimethylthiazol-2-yl)-2,5-diphenyltetrazolium bromide, MTT assay) and genotoxicity (comet assay), to make sure that the plant parts were safe for human use. This was done as follows:

1. Stock extract preparation. A powder sample was mixed with ethanol at a ratio of 1 g:5 mL and soaked for 72 h. Each solution was filtered through Whatman No.1 filter paper. The solvent of the filtrate was removed with a rotary evaporator (Rotavapor R-210, Buchi, Flawil, Switzerland) at 800–1000 mbar, 15 °C and 600 rpm for 2 h. Then, DMSO was added to the extracts until completely dissolved and maintained as stock extracts at −20 °C until the MTT and comet experiments were conducted.
2. Human peripheral blood mononuclear cell (PBMCs) preparation. PBMCs were isolated from sodium heparin anticoagulated venous blood from a blood bank using FicollPaque Plus (GE Healthcare, Chicago, IL, USA). Freshly isolated PBMCs with viabilities of at least 98% were used for the toxicity testing. The cells were suspended at a concentration of 10^6^ cells/mL for MTT assay and 4–6 × 10^5^ cells/mL for comet assay in modified RPMI-1640 medium supplemented with 10% FBS and 1% antibiotic (streptomycin and penicillin).
3. MTT assay. The stock extract was serially 10-fold diluted with water for five working concentrations. The prepared cells were seeded in 96-well plates with 125 µL per well. Then, 12.5 µL of the extract working concentrations were added to the corresponding wells and incubated for 24 h in a humidified CO_2_ incubator (Esco lifescience, Changi, Singapore) at 37 °C and 5% CO_2_. Corresponding DMSO concentrations were similarly prepared as vehicle controls. Untreated cells and UV-treated cells were the negative and positive controls, respectively. After this, the plates were centrifuged at 1500 rpm for 10 min and the medium was removed. MTT (Sigma, St. Louis, MO, USA) was added to a final concentration of 0.5 mg/mL and the plates were wrapped with aluminum foil and incubated for 4 h at 37 °C. Formazan crystals were solubilized by adding 100 µL DMSO to each well. The plates were kept in the dark for 2–4 h. The absorbance was read at 570 nm with a microtiter plate spectrophotometer (Multifunction microplate reader; Molecular devices, San Jose, CA, USA). Wells containing medium and MTT without cells were used as blanks. Each concentration treatment was performed in triplicate. All values were expressed as the mean ± SD. The cellular reduction of MTT formed violet crystal formazan through mitochondrial succinate dehydrogenase activity of the viable cells. The violet crystal formazan was quantified following the methods of Freshney et al. [29]. Percentages of cell viability were calculated using the equation cell viability (%) = average viable treated cells/average viable negative control cells × 100 to reveal the cytotoxicity of the plant extracts. Doses inducing 50% inhibition of cell viability (IC_50_ value) were determined by plotting a graph of the extract concentration against the cell viability. The IC_50_ value was used for the LD_50_ calculation [30] to infer hazardous levels, according to the World Health Organization guidelines [20].
4. Comet assay. The concentration at IC_50_ value or the maximum-treated concentration in the case of no IC_50_ value was used in the comet assay to assess the genotoxicity of plant extracts, following the method described by Singh et al. [31]. Briefly, 500 µL cells in media were added with 50 µL extracts in a 1.5 mL microtube and incubated for 24 h in a humidified CO_2_ incubator at 37 °C and 5% CO_2_. Then, the DNA was checked by electrophoresis. The electrophoresis buffer consisted of 0.3 M NaOH and 1 mM EDTA (pH = 10). Power was supplied at a constant of 3.4 v/cm, with an adjustment to 300 mA, for 25 min. To quantify the level of DNA damage, the extent of DNA migration was defined using the Olive Tail Moment (OTM), which is the relative amount of DNA in the tail of the comet multiplied by the median migration distance. The comets were observed at 200× magnifications and images were obtained using an image analysis system (Zeiss, Jena, Germany) attached to a fluorescence microscope (Nikon, Minato-ku, Japan), equipped with a 560 nm excitation filter, 590 nm barrier filter, and a CCD video camera PCO (Kelheim, Germany). At least 150 cells (50 cells for each of the triplicate slides) were examined for each experiment. The CASP software version 1.2.3 (CASPlab, Wroclaw, Poland) was used to analyze the OTM. The negative and positive controls were untreated cells and UV-treated cells, respectively. All experiments were performed in triplicate. The cultures were scored for the experiment. All values were expressed as the median ± S.D. The nonparametric Mann-Whitney U test was used for statistical analyses of the comet assay results; statistical significance was set at *p* < 0.05.

## 5. Conclusions

Mc rawfs contain a new substance, identified as α-EG, along with pinoresinol, which was previously found in sufficient concentrations and quantities to stimulate collagen growth and promote whitening and minimize dull skin, photoaging and wrinkle formation. These two substances are useful for natural product creation in natural and modified forms. They were found to be without toxicity at both cellular and DNA levels. Additionally, the leaves of the plant can be consumed but in limited amounts, i.e., not more than 2000 mg/kg body weight.

## Figures and Tables

**Table 1:** A summary of chemical constituents sorted by relative content percentages, analyzed by GC-MS in hexane and ethanol *Morus alba* (Ma) and *Morinda citrifolia* (Mc) extracts. For Mc, whole fruits (raw with seeds) were used.

Compound Name	INCI Name	CAS Number	Chemical Formula	Relative Content (%)					
				*Morinda citrifolia*				*Morus alba*	
				Fruits		Leaves		Leaves	
				Ethanol	Hexane	Ethanol	Hexane	Methanol	Hexane
3-Hydroxy-2,3-dihydromaltol	-	-	C_6_H_8_O_4_	0.89	-	0.51	-	-	-
Ethyl-α-d-glucopyranoside	-	-	C_8_H_16_O_6_	54.12	-	52.33	-	-	-
Palmitic acid	Palmitic acid	57-10-3	C_16_H_32_O_2_	11.82	-	1.33	-	14.22	19.32
Phytol	Phytol	150-86-7/7541-49-3	C_20_H_40_O	4.39	3.30	13.19	33.26	14.93	16.04
Lanosteryl acetate	-	2671-68-3	C_32_H_52_O_2_	-	-	-	-	-	11.52
Lupeol acetate	-	1617-68-1	C_32_H_52_O_2_	-	-	-	-	-	10.30
dl-α-Tocopherol	Tocopherol	1406-66-2	C_29_H_50_O_2_	1.87	2.61	4.30	-	-	-
Methyl oleanonate	-	-	C_31_H_48_O_3_	3.95	-	2.27	-	-	-
2-Methoxy-5-Vinylphenol	-	-	C_9_H_10_O_2_	-	-	1.56	-	-	-
Ethyl palmitate	Ethyl palmitate	628-97-7	C_18_H_36_O_2_	-	-	0.43	-	-	-
Chloropyrilene	-	-	C_14_H_18_ClN_3_S	-	-	0.43	-	-	-
Glycerol 1,3-dipalmitate	-	-	C_35_H_68_O_5_	-	-	0.52	-	-	-
2-Palmitoylglycerol	-	-	C_19_H_38_O_4_	-	-	1.43	-	-	-
Glycerol 2-monooleate	-	-	C_21_H_40_O_4_	-	-	1.92	-	-	-
Squalene	Squalene	111-02-04	C_30_H_50_	-	-	4.97	-	-	-
Campesterol	-	-	C_28_H_48_O	-	-	2.90	4.02	-	1.41
γ-Sitosterol	Sitosterol	83-46-5	C_29_H_50_O	-	-	4.22	6.24	13.80	10.32
Methyl linolenate	-	112-63-0	C19H32O2	-	-	-	-	4.32	-
Linolenic acid	Linolenic acid	60-33-3	C18H30O2	-	-	-	-	3.45	-
Vitamin E	Tocopherol	1406-66-2	C_29_H_50_O_2_	-	-	1.80	-	2.83	-
Methyl arjunolate	-	-	C_31_H_50_O_5_	-	-	0.86	-	-	-
α-Tocopherolquinone	-	-	C_29_H_50_O_3_	-	-	-	1.91	-	-
Stigmasterol	-	-	C_29_H_48_O	-	-	-	5.97	-	1.39
Benzyl beta-d-glucoside	-	4304-12-5	C_13_H_16_O_7_	-	-	-	-	4.18	-
Aurantiamide	-	58115-31-4	C_25_H_26_N_2_O_3_	-	-	-	-	4.08	-
Octadecanoic acid	-	57-11-4	C_18_H_36_O_2_	-	-	-	-	1.35	2.95
Lupenone	-	1617-70-5	C_30_H_48_O	-	-	-	-	-	2.84
Simiarenol	-	1615-94-7	C_30_H_50_O	-	-	-	-	-	2.83
ethyl 4-ethoxybenzoate	-	23676-09-7	C_11_H_14_O_3_	-	-	-	-	2.05	0.98
Lanosterol	-	79-63-0	C_30_H_50_O	-	-	-	-	-	1.87
Loliolide	-	5989-02-6	C_11_H_16_O_3_	-	-	-	-	1.79	-
Vomifoliol	-	23526-45-6	C_13_H_20_O_3_	-	-	-	-	1.68	-
Phytol acetate	-	10236-16-5	C_22_H_42_O_2_	-	-	-	-	-	1.48
6,10,14-Trimethylpentadecan-2-one	-	502-69-2	C_18_H_36_O	-	-	-	-	1.42	-
Dodecanoic acid	Lauric Acid	143-07-7	C_12_H_24_O_2_	-	-	-	-	-	1.33
Glycerol-beta-palmitate	-	23470-00-0	C_19_H_38_O_4_	-	-	-	-	1.11	-
Dotriacontane	-	544-85-4	C_32_H_66_	-	-	-	-	-	0.94
γ-Tocopherol	-	54-28-4	C_28_H_48_O_2_	-	-	-	-	0.56	0.88
2-Pentadecanone, 6,10,14-trimethyl-	Hexahydrofarnesyl acetone	502-69-2	C_18_H_36_O	-	-	-	-	-	0.82
delta-Tocopherol	-	119-13-1	C_27_H_46_O_2_	-	-	-	-	0.75	-
(2,3-Diphenylcyclopropyl)methyl phenyl sulfoxide, trans-	-	131758-71-9	C_22_H_20_OS	-	-	-	-	-	0.63
Unknown	-	-	-	22.96	94.09	5.03	48.60	27.48	12.15

**Table 2:** Pinoresinol and α-EG measurements by GC compared to pinoresinol and α-EG standards from *Morinda citrifolia* (Mc) plant parts.

Plant Samples	Filtrate Volume (mL)		Peak Area (pA × s)		Concentration (mg/mL)		Amounts (mg/g Sample)	
	Pinoresinol	α-EG	Pinoresinol	α-EG	Pinoresinol	α-EG	Pinoresinol	α-EG
Leaves	4.50	10.00	49.1908	62.1647	0.09	0.30	0.20	1.51
Unripe fruit pulp	5.20	31.00	78.4832	17.0516	0.13	0.11	0.34	1.75
Ripe fruit pulp	5.30	34.00	89.0466	24.9789	0.14	0.15	0.38	2.48
Raw fruit with seeds	5.00	27.00	65.4722	56.9494	0.11	0.28	0.28	3.77
Seeds	5.20	26.00	38.0631	7.3699	0.08	0.07	0.20	0.94

**Table 3:** Comparative values of stimulatory expression with and without *Morus alba* (Ma) protein extracts added to *Morinda citrifolia* (Mc) parts contains α-EG and pinoresinol; these mixtures were expected to yield higher expression values.

Treatment and Control	Conc. (mg/mL)	Human Dermal Fibroblast Cells										
		Ct Value of *GADPH*	Gene Expression Value (%)									
			*COL1A1*		*COL1A2*		*COL3A1*		*FGF1*		*FGF7*	
			−	+Ma	−	+Ma	−	+Ma	−	+Ma	−	+Ma
Control	-	18.70	190	70	110	100	120	100	160	90	210	110
Mc unripe fruit pulp	6.50, 3.11	34.45	540	2450	960	1360	440	240	390	790	750	1960
Mc raw fruits with seeds	5.06, 3.11	37.19	410	3200	530	960	2870	1130	970	2670	2150	660
Mc seeds	1.04, 3.11	34.88	2030	1810	2930	200	970	320	170	530	2700	140

**Table 4:** Comparative values of inhibition expression with and without *Morus alba* (Ma) protein extract added to *Morinda citrifolia* (Mc) parts containing α-EG and pinoresinol.

The Mixture Samples at a Ratio 6:4	Conc. (mg/mL)	Human Dermal Fibroblast Cells								
		Ct Value of *GADPH*	Gene Expression Value (%)							
			*TYR*		*α-MSH*		*NRF2*		*HLE*	
			−	+Ma	−	+Ma	−	+Ma	−	+Ma
Control	-	21.20	116.90	53.20	55.70	106.20	115.40	109.40	10.50	66.00
Mc leaves + Ma leaves	3.90, 3.11	36.90	0.20	8.70	2.60	6.40	3.80	1.40	0.20	5.30
Mc raw fruits with seeds + Ma leaves	5.06, 3.11	37.20	0.10	11.00	6.40	1.90	4.90	4.80	1.20	8.40
Mc seeds + Ma leaves	1.04, 3.11	35.70	5.40	3.90	29.80	8.20	5.80	3.40	0.50	11.80

**Table 5:** Comparative values of inhibition expression with and without *Morus alba* (Ma) protein extract added to *Morinda citrifolia* (Mc) parts containing α-EG and pinoresinol.

Plant Samples	Working Conc. (mg/mL)	IC_50_ (mg/mL)	LD_50_ (mg/kg rat)	% Cell Viability (Mean ± S.D.)	Olive Tail Moment (Median ± S.D.)	*p*-Values
Negative control	-	-	-	-	0.0972 ± 0.0897	-
Mc leaves	3.90	3.00	2077.25	33.82 ± 0.04	9.9719 ± 0.6977	<0.0001
Mc raw fruits with seeds	5.06	-	-	62.17 ± 0.04	0.1093 ± 0.0891	0.1652
Mc leaves + Ma leaves	3.90, 3.11	3.10	2102.74	37.44 ± 0.08	7.1208 ± 0.5955	<0.0001
Mc raw fruits with seeds + Ma leaves	5.06, 3.11	4.20	2354.23	39.74 ± 0.04	3.2936 ± 0.1640	<0.0001

## Data Availability

Not applicable.

## Abbreviations

Mc	*Morinda citrifolia*
α-EG	ethyl-α-d-glucoside
Pinoresinol	3,3′-bisdemethylpinoresinol
ufp	unripe fruit pulp
rfp	ripe fruit pulp
rawfs	raw fruits with seeds
*NHDF*	normal human dermal fibroblast
qRT-PCR	quantitative reverse transcription-polymerase chain reaction
Ma	*Morus alba*
PBMCs	human peripheral blood mononuclear cells
